# Supplementary material for: Aromatic Hydrocarbon Removal by Novel Extremotolerant Exophiala and Rhodotorula Spp. from an Oil Polluted Site in Mexico
Source: J Fungi (Basel). 2020 Aug 14;6(3):135. doi: 10.3390/jof6030135 (PMC7559356; doi:10.3390/jof6030135)
Supplement: Supplementary file 1 [file jof-06-00135-s001.zip › jof-892282-supplementary.docx]

Supplementary Material

Table S1. Comparison of hydrocarbon degradation by different *Exophiala* and *Rhodotorula* strains. Conditions as temperature, media, aeration, etc. are not the same between the different works. In some articles other hydrocarbons were also tested (anthracene, for example).

| Strain | Compound/concentration | % removal | Reference |
| --- | --- | --- | --- |
| *Exophiala* sp. UTMC 5043 | Phenanthrene  100 ppm | 89 | Farahnaz, et al., 2018 |
| *Exophiala macquariensis* | Toluene  80 ppm | 50 | Zhang, et al, 2019 |
| *Exophiala. oligosperma* | Toluene  50 ppm | 100 | Estevez et al, 2005 |
| *Rhodotorula mucilaginosa* A29 | Toluene  150 ppm | 88 | Hesham et al 2018 |
| *Rhodotorula diobovatum* | Toluene  150 ppm | 85 | Hesham et al 2018 |
| *Rhodotorula glutinis* | Toluene  200ppm | 47 | Romero et al., 1998 |
